# Synthesis of Molecularly Imprinted Polymers for Amino Acid Derivates by Using Different Functional Monomers

**DOI:** 10.3390/ijms12031735

**Published:** 2011-03-07

**Authors:** Sonia Scorrano, Lucia Mergola, Roberta Del Sole, Giuseppe Vasapollo

**Affiliations:** Dipartimento di Ingegneria dell’Innovazione, Università del Salento, via per Arnesano Km 1, 73100 Lecce, Italy; E-Mails: lucia.mergola@unisalento.it (L.M); roberta.delsole@unisalento.it (R.D.S.)

**Keywords:** molecularly imprinted polymer (MIP), Fmoc-3-nitrotyrosine (Fmoc-3NT), 2-vinylpyridine (2-VP), recognition mechanism

## Abstract

Fmoc-3-nitrotyrosine (Fmoc-3-NT) molecularly imprinted polymers (MIPs) were synthesized to understand the influence of several functional monomers on the efficiency of the molecular imprinting process. Acidic, neutral and basic functional monomers, such as acrylic acid (AA), methacrylic acid (MAA), methacrylamide (MAM), 2-vinylpyridine (2-VP), 4-vinylpyridine (4-VP), have been used to synthesize five different polymers. In this study, the MIPs were tested in batch experiments by UV-visible spectroscopy in order to evaluate their binding properties. The MIP prepared with 2-VP exhibited the highest binding affinity for Fmoc-3NT, for which Scatchard analysis the highest association constant (2.49 × 10^4^ M^−1^) was obtained. Furthermore, titration experiments of Fmoc-3NT into acetonitrile solutions of 2-VP revealed a stronger bond to the template, such that a total interaction is observed. Non-imprinted polymers as control were prepared and showed no binding affinities for Fmoc-3NT. The results are indicative of the importance of ionic bonds formed between the –OH residues of the template molecule and the pyridinyl groups of the polymer matrix. In conclusion, 2-VP assists to create a cavity which allows better access to the analytes.

## 1. Introduction

Molecularly imprinted polymers (MIPs) are a promising alternative to provide tailor-made receptor binding sites via the rearrangement of the template and functional monomer [1–4]. Molecularly imprinted technique is based on a process where functional monomer and cross-linker are copolymerized in the presence of the template molecules, involving the formation of cavities in which the template is arranged. In a first step, the template interacts with a functional monomer that contains complementary functional groups through hydrogen bonding, reversible covalent bonds, electrostatic interactions, and van der Waals forces. In a second step, the monomer-template complex is polymerized in the presence of a large excess of the cross-linking agent. The chemical bonds between the monomer and the cross-linker establish the position of the functional monomer around the template. Finally, after polymerization, the template can be removed from the polymeric structure revealing binding sites with shape, size and chemical functionality complementary to it [5,6].

The synthesis process is easy, low-cost and the resulting polymers are stable, versatile and resistant to a wide range of pHs, solvents and temperature. These properties allow MIPs to be applied to a wide range of template including amino acids, amino acid derivates, peptides, proteins, nucleotides, drugs, environmental pollutants.

The literature supplies many examples of artificial receptors that recognize amino acids so the development of synthetic receptors for amino acids and their derivates is greatly increasing nowadays [7–9]. For this study, Fmoc-L-3-nitrotyrosine (Fmoc-3NT), a N^α^-protected amino acid, was chosen as template to synthesize a highly selective MIP. The protected group is fundamental to prevent the formation of electrostatic interaction between the terminal charged group on the template and the opposed charged group on the functional monomer [10].

**Figure 1.**
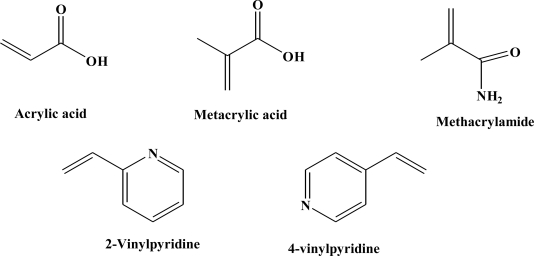
Structures of commercially available functional monomers used for imprinting optimization.

Several aspects, including temperature, solvent, functional monomer, cross-linking density, synthesis conditions, template morphology and size, define the porous structure and the surface area of the MIP and were thus studied in polymer preparation [11,12]. Also, it has been seen that the molecule selectivity and the binding properties of the MIP are strongly influenced from the functional monomers. Considering this, in the last 20 years, the use of many functional monomers has been studied in non-covalent imprinting [7,13,14]. Since different monomers have been employed in preparation of MIPs towards a wide range of templates, the number of functional monomers available is increasing [15]. Furthermore, because the influence of functional monomers in the formation of the monomer-template complexes is an important key in the creation of binding sites, in the present work, acidic, neutral and basic functional monomers, such as acrylic acid (AA), methacrylic acid (MAA), methacrylamide (MAM), 2-vinylpyridine (2-VP) and 4-vinylpyridine (4-VP) (Figure 1) have been chosen to test and define the most successful functional monomer. In this way it was possible to investigate which functional monomer establishes most interactions with the template and to develop the best procedure of synthesis.

We prepared different imprinted polymers for Fmoc-3NT using acetonitrile as porogen and ethylene glycol dimethacrylate (EGDMA) and 2,2′-azobisisobutyronitrile (AIBN) as cross-linker and initiator, respectively. Five different MIPs were prepared, using different functional monomers: MIP1 with AA, MIP2 with MAA, MIP3 with MAM, MIP4 with 2-VP and MIP5 with 4-VP. The corresponding un-imprinted blank polymers were prepared using the same conditions but in the absence of Fmoc-3NT. The MIPs prepared were tested in batch experiments by UV-vis spectroscopy and the binding characteristics of the MIPs were examined by Scatchard analysis.

## 2. Results and Discussion

The synthesis of MIPs selective for amino acid derivates, studied as tumor markers, is an important field of research today. In order to investigate the binding mechanism of MIPs for an amino acid derivate (Fmoc-3NT) under different reaction conditions, a range of imprinted polymers with different functional monomers (AA, MAA, MAM, 2-VP, 4-VP) have been prepared. It was seen that the polymer’s affinity is directly related to the strength of the complex formed between template and monomer, so the choice of functional monomer is very important for the molecular recognition of MIP. All prepared polymers were tested in batch rebinding experiments and the binding behavior for Fmoc-3NT was evaluated. The binding data were processed with Scatchard equation in order to estimate the binding properties of the polymers.

**Figure 2.**
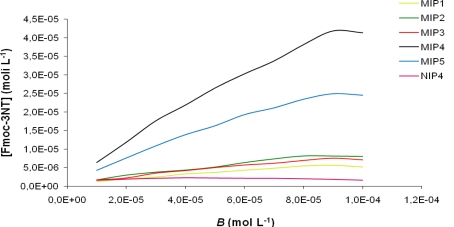
Binding isotherm for MIP1, MIP2, MIP3, MIP4, MIP5 and NIP4.

The binding curves showing the amount of Fmoc-3NT bound (*B*) in 18 h (in a batch method) as a function of the initial concentration of Fmoc-3NT for all synthesized MIPs is shown in Figure 2. MIP4, prepared using 2-VP as functional monomer, exhibited the highest binding affinity for Fmoc-3NT, compared to the other MIPs. All NIPs showed a similar behavior with a very low binding affinity. As a reference NIP4 binding isotherm was reported in Figure 2.

**Figure 3.**
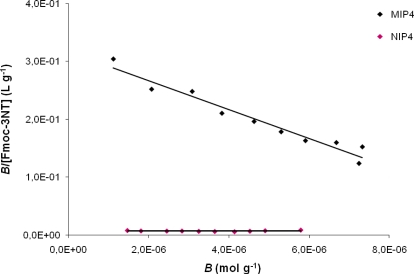
Scatchard plot for MIP4 and NIP4.

Figure 3 shows the Scatchard plot for MIP4 and NIP4. There is a single straight line, which indicates that one kind of binding site is populated in the MIP. This fact is very interesting since a non linear profile was commonly observed in the Scatchard assessment of MIPs indicating the presence of binding sites that exhibit various affinities to the ligand.

The properties of the binding sites in MIP1, MIP2, MIP3, MIP4 and MIP5 are summarized in Table 1. As can be seen, the MIP4 showed the highest binding ability for Fmoc-3NT, for which Scatchard analysis the highest K_a_ (2.49 × 10^4^ M^−1^) has been obtained.

Carboxylic acid-based monomers are the most widely applied in non-covalent molecular imprinting, but in this work, the MIPs prepared using basic monomers, such as 2-VP and 4-VP, exhibited higher binding affinities. This is probably due to the fact that the basic functional monomers usually interact strongly with some templates [3]. AA and MAA interact with Fmoc-3NT via hydrogen bonds such as MAM. On the other hand, basic monomers establish ionic interactions with amino acid, which are stronger than hydrogen bonds in polar solvents, such as acetonitrile; this porogen has been chosen, in this study because most amino acids derivates are more soluble in it. For this reason, in MIP1, MIP2 and MIP3 the binding capacity is weaker than in MIP4 and MIP5. The non-imprinted polymers showed no binding affinities for Fmoc-3NT, confirming that the binding capacity of the imprinted polymers for this molecule is due to the imprinting of the polymer matrix and not to the intrinsic affinity of the template to the functional monomer.

**Figure 4.**
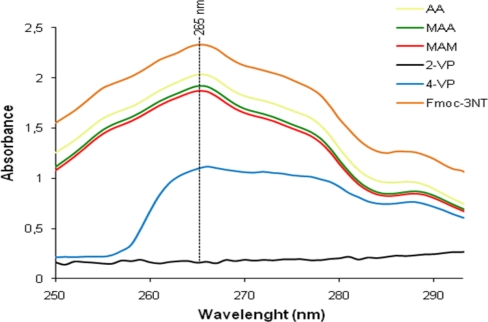
Changes of the titration spectra of Fmoc-3NT with the addition of different monomers.

The ability of the different functional monomers to bind Fmoc-3NT was investigated also using UV-vis absorption method that is widely used for its high sensitivity to host-guest binding. UV-visible titration of AA, MAA, MAM, 2-VP and 4-VP with Fmoc-3NT was conducted at 265 nm in acetonitrile at 23 °C. Typical spectral changes upon the addition of Fmoc-3NT to each monomer are shown in Figure 4. As can be seen, the addition of Fmoc-3NT to each monomer resulted in a gradual increase of the characteristic absorptions of the template molecule. Titration of Fmoc-3NT into acetonitrile solution of AA, MAA and MAM revealed no appreciable change in the absorption spectra of the amino acid, implying that those functional monomers do not interact significantly with the template. On the other hand, titration of Fmoc-3NT into acetonitrile solutions of the pyridinyl monomers revealed a stronger bond with the template, especially with 2-VP in which a total interaction can be observed.

The better performance of the MIPs synthesized with pyridinyl monomers indicates that ionic bonds are formed between the –OH residues of the template molecule and the pyridinyl residues of the polymer. However, 2-VP and 4-VP bind similarly to template but the justaposition of the functional group and the interactive amine group could influence the steric interaction of the template with the structure of the binding site. Thus 2-VP, used to synthesize the MIP4, helps to form a cavity which allows better access to the analytes.

## 3. Experimental Section

### 3.1. Reagents and Apparatus

Fmoc-L-3-nitrotyrosine (Fmoc-3NT), ethylene glycol dimethacrylate (EGDMA), acrylic acid (AA), methacrylamide (MAM), 2-Vinylpyridine (2-VP), 4-vinylpyridine (4-VP) and acetic acid were purchased from Sigma-Aldrich (Steinheim, Germany). Methacrilylic acid (MAA) and α-α’-azoisobutyronitrile (AIBN) were supplied from Fluka (Steinheim, Germany). Analytical grade acetonitrile (MeCN) and methanol (MeOH) were obtained from J.T. Baker (Deventer, Holland). Batch rebinding experiments were carried out using a Cary 100 Scan UV-visible spectrophotometer (Varian, Palo Alto, CA, USA).

### 3.2. Preparation of Molecularly Imprinted Polymers

**Figure 5.**
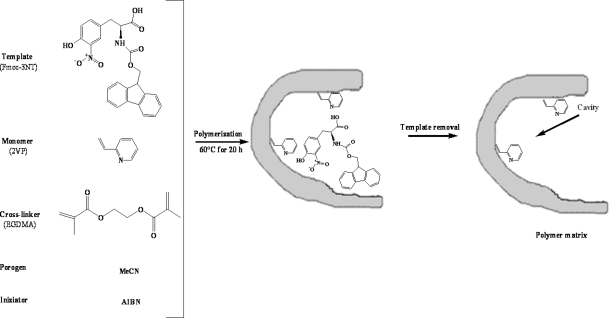
Schematic representation of the molecular imprinting of Fmoc-3NT using 2-VP as functional monomer.

Fmoc-3NT-imprinted polymers were synthesized by radical polymerization (Figure 5). The template, Fmoc-3NT (0.0468 mmol), was dissolved in about 1 mL of acetonitrile in a glass tube, using a volume of porogen corresponding to 4/3 of the total amount of cross-linker and monomer. 0.665 mmol of functional monomer (AA, MAA, MAM, 2-VP, 4-VP) and 3.520 mmol of EGDMA were added to the solution. Subsequently, 0.042 mmol of AIBN as initiator was added in the mixture. The solution was purged with nitrogen gas for 5 min in a sonicating bath to remove oxygen and thermal polymerization was performed at 60 °C for 20 h. Five MIPs synthesized under the same conditions and varying only by their composition as shown in Table 2, were obtained.

After the polymerization, the resultant bulk polymer was ground in a mortar and sieved. The polymer was exhaustively washed with methanol/acetic acid (7/3, v/v) to remove the template molecule and then with methanol to remove the residual acetic acid. Finally, the resulting particles were dried under vacuum and used for rebinding studies. We also prepared non-imprinted polymers as control, following the same procedure described above, except for the addition of the template molecule (Fmoc-3NT).

### 3.3. UV-Visible Titration Experiments

A series of suspensions in acetonitrile (5 mL), each containing 0.665 mmol of the different functional monomers (AA, MAA, MAM, 2-VP, 4-VP) were prepared. Small aliquots of the solution of Fmoc-3NT (0.0468 mmol) in acetonitrile at 23 °C, were added to each solution of monomer and was measured the absorbance at 265 nm. The changes in absorbance and difference absorption spectra of these solutions were determined using only the functional monomers in acetonitrile as reference. The effects of all monomers by the addition of Fmoc-3NT solutions on the absorption values were taken into account during analysis.

### 3.4. Batch Rebinding Experiments and Scatchard Analysis

For adsorption isotherms, 20 mg of each polymer was incubated in 3.5 mL of Fmoc-3NT in acetonitrile spanning the concentration range from 0.1× 10^−4^ M to 1 × 10^−4^ M. The suspension was shaken for 18 h at room temperature. Then, the polymer was removed by filtration and the resulting solution was analyzed by UV-vis spectrophotometer at 265nm. The amount of Fmoc-3NT bound to the polymer, *B*, was calculated by subtraction of the concentration of free Fmoc-3NT, [Fmoc-3NT], from the initial Fmoc-3NT concentration. [Fmoc-3NT] was determined as an average value of three measurements. Below we report the Scatchard equation (1) provided in the Scatchard analysis:

(1) B/[Fmoc-3NT] =(Bmax−B)Ka

where B_max_ is the apparent maximum number of binding sites and K_a_ is the association constant. Therefore, B_max_ and K_a_ of the polymer were determined from the slope and the intercept, respectively, by plotting B/[Fmoc-3-NT] *versus* B.

## 4. Conclusions

Molecularly imprinted polymers for amino acid derivates, by using five different functional monomers, were prepared. 2-VP as functional monomer, which can interact with –OH residues of the print molecule, showed best results in binding capacity. Considering these promising results, for easily constructed and highly selective MIPs for amino acid derivates, new investigations are now being directed towards their potential use for analytical purpose. Thus, new MIPs will be synthesized to use as sorbent materials for the purification and pre-concentration of amino acid derivates from complex samples and the development of MIP-based sensors arrays for amino acid derivate discriminations.

## Figures and Tables

**Table 1.** Association constant (*K*_a_) and maximum number of binding sites (*B*_max_) for MIP1, MIP2, MIP3, MIP4 and MIP5.

**Polymer**	*K***_a_ (10^4^ M^−1^)**	*B***_max_ (μM g^−1^)**
MIP1	1.18 ± 0.4	1.80
MIP2	1.21 ± 0.3	2.80
MIP3	1.32 ± 0.5	2.26
MIP4	2.49 ± 0.4	12.65
MIP5	1.58 ± 0.4	8.37

**Table 2.** Composition of the synthesized polymers.

**Polymer**	**MIP1**	**MIP2**	**MIP3**	**MIP4**	**MIP5**
Template (0.0468 mmol)	Fmoc-3NT	Fmoc-3NT	Fmoc-3NT	Fmoc-3NT	Fmoc-3NT
Monomer (0.665 mmol)	AA	MAA	MAM	2-VP	4-VP
Cross-linker (3.520 mmol)	EGDMA	EGDMA	EGDMA	EGDMA	EGDMA
Porogen (μL)	943	957	960	979	979
